# Nlrp3 Prevents Early Renal Interstitial Edema and Vascular Permeability in Unilateral Ureteral Obstruction

**DOI:** 10.1371/journal.pone.0085775

**Published:** 2014-01-15

**Authors:** Wilco P. Pulskens, Loes M. Butter, Gwendoline J. Teske, Nike Claessen, Mark C. Dessing, Richard A. Flavell, Fayyaz S. Sutterwala, Sandrine Florquin, Jaklien C. Leemans

**Affiliations:** 1 Department of Pathology, Academic Medical Center, University of Amsterdam, Amsterdam, The Netherlands; 2 Department of Immunobiology, Yale University School of Medicine and Howard Hughes Medical Institute, New Haven, Connecticut, United States of America; 3 Department of Internal Medicine, University of Iowa, Iowa City, Iowa, United States of America; INSERM, France

## Abstract

Progressive renal disease is characterized by tubulo-interstitial injury with ongoing inflammation and fibrosis. The Nlrp3 inflammasome contributes to these pathophysiological processes through its canonical effects in cytokine maturation. Nlrp3 may additionally exert inflammasome-independent effects following tissue injury. Hence, in this study we investigated potential non-canonical effects of Nlrp3 following progressive renal injury by subjecting WT and Nlrp3-deficient (−/−) mice to unilateral ureter obstruction (UUO).

Our results revealed a progressive increase of renal Nlrp3 mRNA in WT mice following UUO. The absence of Nlrp3 resulted in enhanced tubular injury and dilatation and an elevated expression of injury biomarker NGAL after UUO. Moreover, interstitial edema was significantly elevated in Nlrp3−/− mice. This could be explained by increased intratubular pressure and an enhanced tubular and vascular permeability. In accordance, renal vascular leakage was elevated in Nlrp3−/− mice that associated with reduced mRNA expression of intercellular junction components. The decreased epithelial barrier function in Nlrp3−/− mice was not associated with increased apoptosis and/or proliferation of renal epithelial cells. Nlrp3 deficiency did not affect renal fibrosis or inflammation.

Together, our data reveal a novel non-canonical effect of Nlrp3 in preserving renal integrity and protection against early tubular injury and interstitial edema following progressive renal injury.

## Introduction

Fibroproliferative diseases, including progressive renal disease are a leading cause of morbidity and mortality worldwide [1]. In the kidney, interstitial inflammation and fibrosis are main predictors for the risk of progression towards end-stage renal disease [2]. Progressive renal disease can be characterized by a cascade of pathophysiological processes, including disruption of tubular integrity, infiltration of inflammatory cells, tubular atrophy, accumulation of (myo)fibroblasts and an increased deposition of extracellular matrix (ECM) molecules. Eventually, this altogether leads to renal fibrogenesis [3], [4], [5].

The innate immune system may contribute to renal fibrotic processes through the activation of toll-like receptor 4 (TLR4) [6] and TLR9 [7], but not TLR2 [8]. An additional mechanism that may orchestrate renal inflammatory and fibrotic processes is the formation and activation of the intracellular inflammasome that consists of Nlrp3, ASC and caspase-1 [9], [10], [11]. Both experimental and human studies show a detrimental role for Nlrp3 in the development of acute and chronic tubulointerstitial disease [9], [10], [11], [12], [13], [14]. In one of these studies, it was shown that the absence of Nlrp3 led to a classical reduction of caspase-1 activation and maturation of IL-1β and IL-18 and diminished renal inflammation and injury in the late phase of experimental chronic kidney disease [10]. Besides this canonical effect of Nlrp3 on cytokine maturation and renal pathology, it is currently thought that Nlrp3 has additional non-canonical effects upon various types of renal injury as well (for review see [15]). Non-canonical effects of Nlrp3 were shown on the promotion of TGFβ-dependent signaling in renal tubular epithelial cells [16]. Moreover, a direct effect of Nlrp3 was shown on renal epithelium following acute ischemic kidney injury, independently of its classical effects on cytokine maturation [17]. This proposes that Nlrp3 might also exert additional effects following experimental models of progressive renal injury. Hence, in this study we investigated whether Nlrp3 might have additional non-canonical functions in kidney homeostasis during the initial phase of progressive renal injury by subjecting wild type and Nlrp3−/− mice to unilateral ureter obstruction (UUO). This study revealed a novel and important function of Nlrp3 in preventing early renal interstitial edema and vascular permeability during UUO.

## Materials and Methods

### Ethics Statement

The Animal Care and Use Committee of the University of Amsterdam approved this study and all included animal experiments (study# DPA101016). Experiments have been conducted according to national guidelines.

### Mice

Pathogen-free 8−12 week old female wild type C57Bl/6 mice were purchased from Charles River Laboratories. Nlrp3−/− mice were generated as described previously [18], backcrossed nine times to a C57Bl/6 background and bred in the animal facility of the Academic Medical Center in Amsterdam, the Netherlands. Only age and sex-matched mice were used in experiments. Nlrp3 deficiency was genetically confirmed by RTPCR screening.

### Unilateral Ureter Obstruction

Unilateral ureter obstruction was induced as described previously [19]. Briefly, all mice received preoperative analgesia (subcutaneous injection of 50μg/kg buprenorphin (Temgesic, Shering-Plough)) and the right ureter was subsequently ligated with 6.0 silk through a small abdominal incision under 2.0% isoflurane-induced anesthesia. The abdomen was closed in two layers and mice were allowed to recover from surgery for 12 hours at 28°C in a ventilated stove. Mice (n = 7/group) were sacrificed 1, 3, 7 and 14 days after surgery. As we demonstrated before, there are no basal differences between wild type and Nlrp3−/− mice that only received anesthetics plus sham-surgery with respect to tubular histology, renal function and inflammation [9]. In the current study the contralateral non-obstructed kidney (t = 14) served as control in order detect potential obstruction-independent compensatory effects due to Nlrp3 deficiency.

In a separate experiment, wild type and Nlrp3−/− mice (n = 4/group) were subjected to UUO as described above. After 1 day, mice received a single intravenous bolus injection of 100µg fluorescein-labeled Dextran (500,000 MW; Life Technologies, D-7136) in a total volume of 100µl or vehicle (saline), 5 minutes before being sacrificed.

### Measurement of mRNA expression

Total RNA was extracted from thirty 20μm frozen total renal tissue sections of each animal or from harvested cells with Trizol reagent (Invitrogen) according to the manufactures protocol. All RNA samples were quantified by spectrophotometry and stored at −80°C until processed for reverse transcription. RNA was converted to cDNA by using oligo-dT as primer. Nlrp3, ASC, caspase-1, NGAL, Claudin-1, -2, -5 and VE-Cadherin mRNA expression was analyzed by real-time quantitative reverse transcription-PCR (RT-PCR) performed on a Roche light cycler with SYBR green PCR master mix. Specific gene expression was normalized to mouse hypoxanthine-guanine-phosphoribosyltransferase (HPRT) house keeping gene expression. SYBR green dye intensity was analyzed with linear regression analysis. Six animals per group were analyzed by real-time quantitative RT-PCR.

### Determining tubular injury and interstitial edema

Renal tissues were fixed in 10% formalin and embedded in paraffin. All histopathological scorings were made in the cortex using PAS-D stained renal sections and performed on coded slides. The percentage of tubular injury (criteria: epithelial flattening, tubular dilatation and brush border loss) and the degree of interstitial edema was estimated by a pathologist in a blinded fashion using a 4-point scale in ten randomly chosen, non-overlapping fields (200x magnification). Degree of injury was graded onto a scale from 0 to 4: 0 = normal; 1 = mild, involvement of less than 25% of the cortex; 2 = moderate, involvement of 25 to 50% of the cortex; 3 = severe, involvement of 50 to 75% of the cortex; 4 = extensive damage involving >75% of the cortex. Degree of edema was graded according: 0 = normal; 1 = mild edema; 2 = moderate; 3 = severe; 4 = extensive edema formation. For visualization of proximal tubular brush borders, renal sections were boiled in 10 mM Tris/1 mM EDTA (pH 9.0) and exposed to rabbit-anti-sodium/glucose transporter-1 (SGLT1) antibodies (Abcam), followed by power rabbit poly-HRP incubation and subsequently developed using 1% H_2_O_2_ and DAB (Sigma-Aldrich) in 0.05 M Tris-HCl (pH 7.9).

### Detection of apoptotic and proliferating tubular epithelial cells

Antigen retrieval was performed by boiling paraffin-embedded tissue sections for 10min in either 10 mM Tris/1 mM EDTA (apoptosis) or sodium citrate buffer (pH 6.0) (proliferation), followed by blocking endogenous peroxidase activity. Slides were incubated with rabbit anti-human active caspase-3 polyclonal antibody (Cell Signaling Technology) or rabbit anti-Ki67 antibody (Sp6; Neomarkers). After incubation with the appropriate secondary antibodies, slides were developed as described above, and counterstained with methyl green (Sigma). To evaluate the degree of apoptosis and proliferation, caspase-3- and Ki67-positive tubular epithelial cells were counted in at least 10 non-overlapping high power fields (magnification 400x). Since tubular atrophy was very severe 14 days post-UUO in all groups, identification of epithelial cells was difficult. Therefore, the total amount of positive stained cells was quantified for this time point. Staining specificity was confirmed by incubating renal slides without the primary antibody, which revealed completely negative sections.

### Detection of renal macrophage and fluorescein-labeled Dextran accumulation

Paraffin-embedded renal sections were deparaffinized and boiled for 10 minutes in 10 mM sodium citrate buffer (pH 6.0) for antigen retrieval. Subsequently, endogenous peroxidase activity and non-specific binding were blocked and slides were exposed to rat anti-mouse F4/80 IgG2b mAb (Serotec) or rabbit anti-FITC (DakoCytomation) antibodies. For macrophage detection, slides were subsequently incubated with rabbit-anti-biotin (Dako), followed by streptavidin-ABC solution (Dako), and for FITC detection slides were incubated with HRP-conjugated goat-anti rabbit IgG (PowerVision; ImmunoVision Technologies Co.). Finally, slides were developed as described above and counterstained with methyl green. The percentage of positive renal F4/80 or FITC-labeled Dextran staining was quantified digitally using Image Pro Plus software version 5.0.

### Detection of fibrosis

For staining of collagen, slides were incubated with 0.2% Picro Sirius Red solution (pH 2.0) for 1 hour followed by incubation within 0.01 M HCL. For myofibroblast staining antigen retrieval was achieved by boiling for 10 minutes in 10 mM sodium citrate buffer (pH 6.0). Subsequently, slides were exposed to mouse anti-human αSMA-IgG2a (DAKO), followed by incubation with goat anti-mouse IgG2a-HRP (Southern Biotech). Slides were developed as described above and counterstained with methyl green. The percentage of positive αSMA and Picro Sirius Red was quantified digitally using Image Pro Plus software version 5.0.

### Preparing kidney homogenate

For cytokine measurements, snap-frozen kidneys were homogenized in Greenberger Lysis buffer (150 mM NaCl, 15 mM Tris, 1 mM MgCl ·H_2_O, 1 mM CaCl_2_ and 1% Triton-X), and incubated for 30 minutes at 4°C. Homogenates were subsequently centrifuged at 14,000rpm for 10 minutes after which supernatants were stored at −80°C until ELISAs were performed. To determine protein content, Bradford Protein Assay (Bio Rad) was used with IgG as standard.

### ELISA

Cytokines and chemokines (Keratinocyte Chemoattractant (KC), Monocyte Chemoattractant-1 (MCP-1), Interleukin 1-β (IL1-β), Tumor Necrosis Factor (TNF-α) and total transforming growth factor-β (TGF-β)) were measured in the kidney homogenates using specific ELISA’s (R&D systems) according the manufactures protocol. Activation of latent TGF-β was done according to the protocol supplied by the manufacturer. The detection limits were 12 pg/ml (KC), 6 pg/ml (MCP-1), 31pg/ml (IL1-β), 31 pg/ml (TNF-α) and 31 pg/ml (TGF-β).

### Characterization of cellular inflammasome component expression

To identify expression of inflammasome components Nlrp3, ASC and caspase-1 in various cell types, wild type primary tubular epithelial cells (pTECs) and primary myofibroblasts were isolated from naïve C57Bl/6 mice and cultured as described before [6]. In addition, immortalized mouse peritubular capillary endothelial cells (IETs;[20]) and wild type bone-marrow derived macrophages (BMDM; LPS+IFNγ stimulated) were harvested and cultured as described before. Cells were stimulated with 10 ng/ml recombinant TGFß (R&D systems) or vehicle (PBS) for 24 hours after which RNA was isolated using Trizol reagent. Cell-type specific gene expression was determined using RT-PCR as described above.

### Statistics

Differences between groups were analyzed using Mann-Whitney U test. Values are expressed as mean ± standard error of the mean (SEM). A value of p<0.05 was considered as statistically significant.

## Results

### Elevated Nlrp3 mRNA expression upon UUO

**Figure 1:**
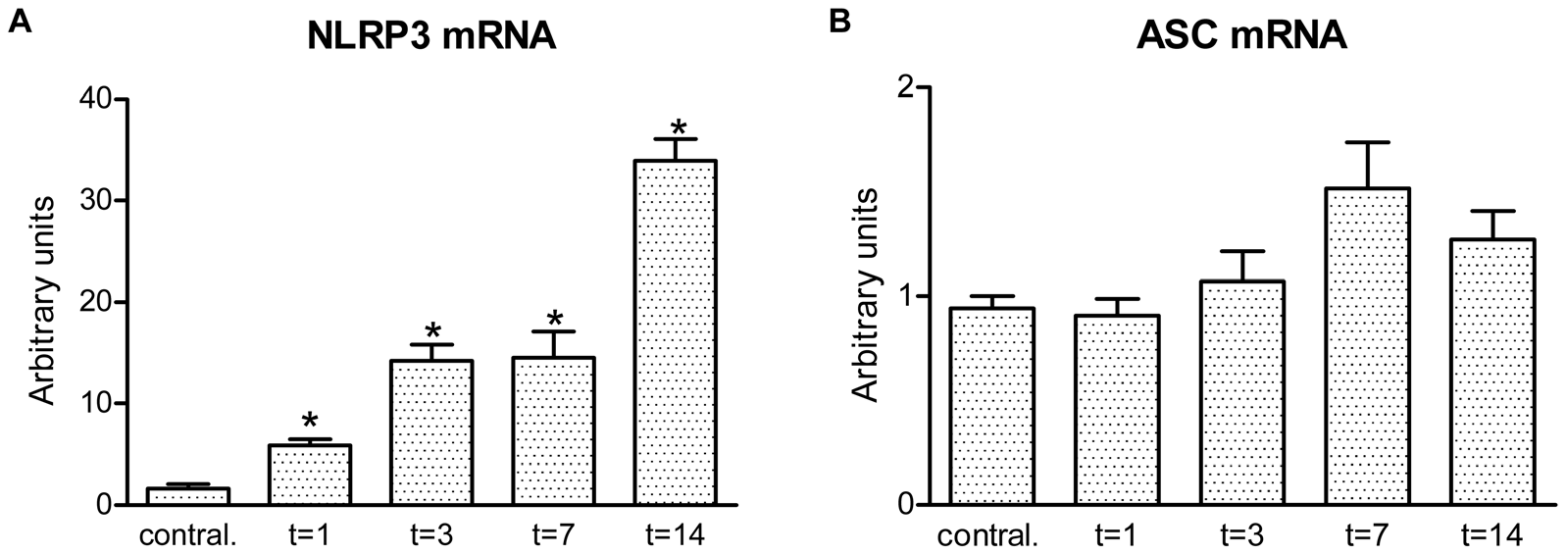
Progressive renal injury enhances renal Nlrp3, but not ASC mRNA expression. Relative expression levels of Nlrp3 (A) and ASC (B) mRNA in total kidney specimen following progressive renal injury. Nlrp3 mRNA expression was significantly enhanced 1, 3, 7 and 14 days post-UUO compared with contralateral kidneys, whereas ASC mRNA remained constitutively expressed. Data are mean±SEM of 6 mice per group. *:p<0.05.

To determine whether progressive renal injury influences expression kinetics of Nlrp3 and ASC, we quantified mRNA levels in total kidney specimens at several time points post-UUO. In line with previous results [10], renal Nlrp3 mRNA levels were significantly enhanced 1, 3, 7 and 14 days post-UUO, when compared with contralateral kidneys (figure 1A). In contrast, ASC mRNA levels remained constitutively present (figure 1B). These data suggest a potential role for Nlrp3 in the underlying pathophysiology of UUO. Characterization of renal non-immune cell types that express inflammasome components revealed that renal tubular epithelial cells and myofibroblasts in particular express Nlrp3, ASC and caspase-1 mRNA. Endothelial cells in contrast hardly displayed Nlrp3 or ASC expression, while caspase-1 is present (figure S1).

### Nlrp3 attenuates renal injury in UUO

**Figure 2:**
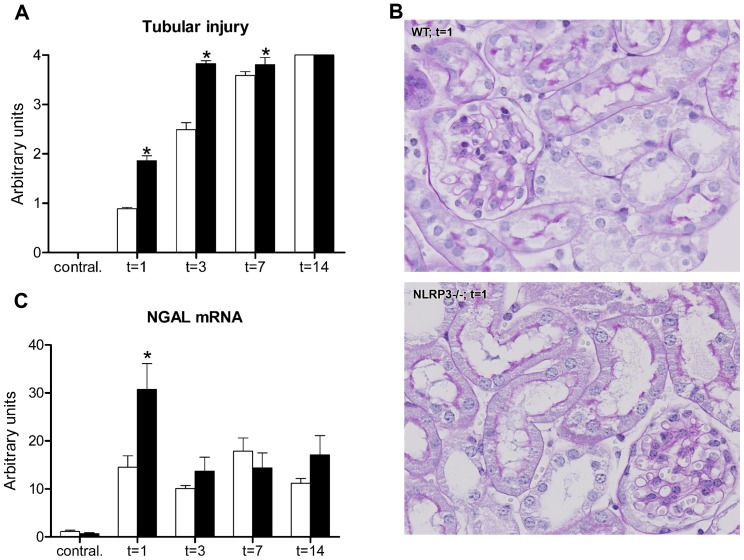
Nlrp3 deficiency increases renal injury following UUO. (A) Semi-quantitative tubular injury score in wild type (white bars) and Nlrp3−/− (black bars) mice following UUO. Nlrp3−/− mice developed significantly more tubular injury when compared to their respective wild type mice 1, 3, and 7 days post-UUO. (B) Representative PASD microphotographs of renal tissue subjected to 1 day of UUO, with apparent interstitial edema in the Nlrp3−/− kidneys (magnification x200). (C) Nlrp3−/− mice displayed enhanced mRNA expression of the renal injury biomarker NGAL compared to their wild type mice following UUO. Data are mean±SEM of 7 mice per group. Contral.  =  contralateral kidneys of mice subjected to 14 days of UUO. *:p<0.05.

We next defined the role of Nlrp3 in progressive renal injury by subjecting wild type and Nlrp3−/− mice to UUO. No abnormalities in tubular histology were observed in naïve Nlrp3−/− kidneys compared to wild type kidneys (data not shown). Following UUO, wild type mice demonstrated a progressive increase in the level of tubular injury, including epithelial flattening, brush border loss and tubular dilatation which reached a maximum at day 14 (figure 2A). Interestingly, Nlrp3−/− mice developed significantly more severe tubular injury 1, 3 and 7 days post-UUO, while after 14 days both genotypes demonstrated maximal score of injury. As shown in representative microphotographs interstitial edema was more pronounced in Nlrp3−/− mice 1 day post-UUO compared to wild type mice (figure 2B). Indeed, semi-quantitative score of the degree of interstitial edema revealed higher levels in obstructed kidneys of Nlrp3−/− mice compared to wild type mice (1.78±0.19 vs. 2.25±0.18 arbitrary units at t = 1; p = 0.008). Additionally, NGAL mRNA was measured as an early and sensitive biomarker of renal injury. Nlrp3−/− mice demonstrated enhanced NGAL expression in their obstructed kidneys 1 day post-UUO compared to wild type mice (figure 2C), while no differences were observed at later time points when tubuli become severely atrophic. Furthermore, enhanced tubular dilatation in Nlrp3−/− kidneys 1 day post-UUO was strengthened by the diffusing and widening expression pattern observed for the apical brush border marker SGLT1 within the proximal tubular segments (figure S2).

### Nlrp3 deficiency reduces renal expression of epithelial and endothelial intercellular junction molecules following UUO

**Figure 3:**
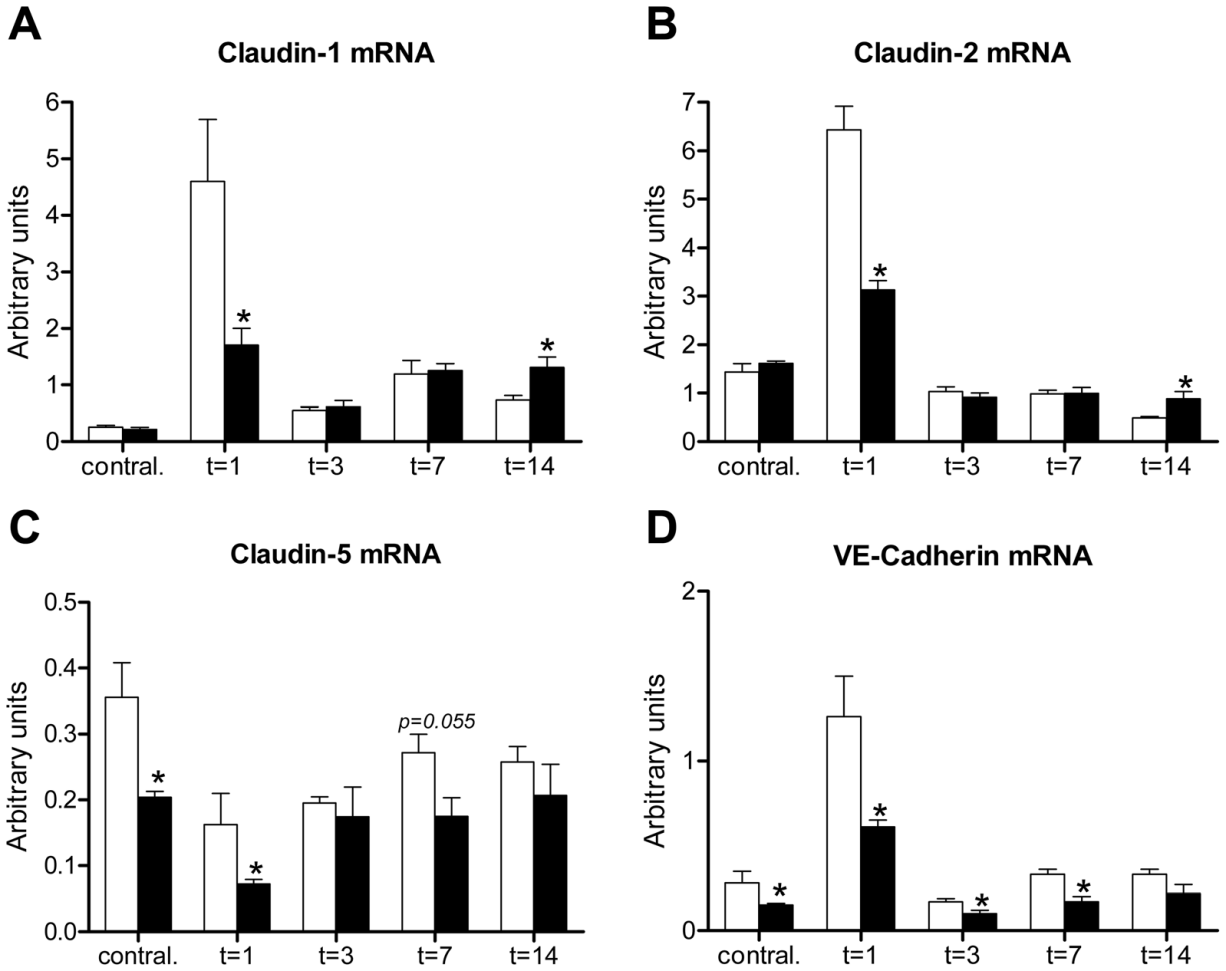
Nlrp3 preserves early expression of renal epithelial- and endothelial-associated Claudins following UUO. Renal mRNA expression of epithelium-associated Claudin-1 (A) and Claudin-2 (B) and vascular endothelium-associated Claudin-5 (C) and VE-Cadherin (D) was reduced in obstructed kidneys of Nlrp3−/− mice (black bars) when compared to wild type mice (white bars) 1 day post-UUO. Whereas VE-Cadherin remained lower, Nlrp3−/− mice displayed a subtle increase in Claudin-1 and -2 expression 14 days post-UUO compared to wild type mice. Contral.  =  contralateral kidneys of mice subjected to 14 days of UUO. Data are mean±SEM of 6 mice per group. *:p<0.05.

In order to investigate whether the enhanced interstitial edema observed in Nlrp3−/− mice might be a consequence of a disturbed epithelial integrity or a leaky microvascular endothelium, we determined the expression of renal Claudin-1, and -2 and Claudin-5 and VE-Cadherin as major constituents composing tight junctions between epithelial and endothelial sheets, respectively. Interestingly, 1 day post-UUO when interstitial edema was most prominent, Nlrp3−/− mice displayed a reduced expression of Claudin-1, -2, -5 and VE-Cadherin in their obstructed kidneys when compared to wild type mice (figure 3A-D). VE-Cadherin expression remained lower in kidneys of Nlrp3−/− mice. In contrast, 14 days post-UUO a subtle increase was observed for Claudin-1 and -2. Claudin-5 and VE-Cadherin mRNA expression was also reduced in contralateral kidneys of Nlrp3−/− mice. These data suggest that Nlrp3−/− mice have less (effective) tight junctions in order to maintain renal epithelial and endothelial integrity following UUO as a consequence of reduced Claudin-1, -2, -5 and VE-Cadherin expression, respectively. This result in combination with increased intratubular pressure as a result of increased dilatation in the Nlrp3−/− mice may contribute to the more severe interstitial edema formation observed in these mice compared to wild type mice. Of note, Nlrp3 deficiency did not affect the basal mRNA expression of these junction components in kidney tissue of naïve sham-mice (figure S3).

### Nlrp3 preserves renal vascular integrity following UUO

**Figure 4:**
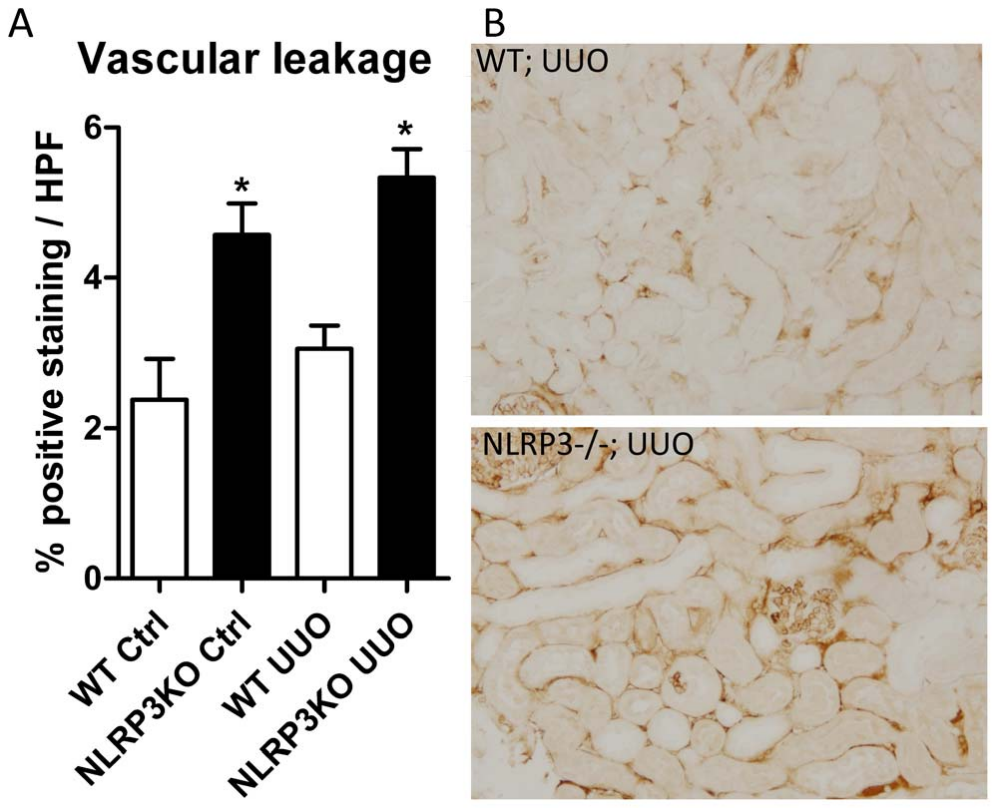
Nlrp3 deficiency elevates vascular leakage in the ligated and non-ligated kidney following UUO. (A) The accumulation of interstitial FITC-Dextran was significantly elevated in both obstructed (UUO) and contralateral (Ctrl) kidneys of Nlrp3−/− mice compared to wild type mice 1 day post-UUO. (B) Representative microphotographs of renal tissue subjected to 1 day UUO, demonstrating elevated interstitial FITC-Dextran accumulation in Nlrp3−/− obstructed kidneys (magnification 200x). Data are mean±SEM of 4 mice per group. *:p<0.05.

To investigate whether Nlrp3 deficiency affects early vascular permeability, wild type and Nlrp3−/− mice were systemically administered high molecular weight FITC-Dextran or vehicle 1 day post-UUO. Interestingly, significant higher amounts of interstitial Dextran were observed in obstructed kidneys of Nlrp3−/− mice compared to wild type mice, suggesting increased vascular leakage in Nlrp3−/− obstructed kidneys (figure 4). No interstitial staining appeared when saline was injected (data not shown). Interestingly, interstitial Dextran accumulation was also elevated in the contralateral kidney of Nlrp3−/− mice compared to wild type mice.

### Nlrp3 deficiency reduces apoptosis, but not proliferation of TECs

**Figure 5:**
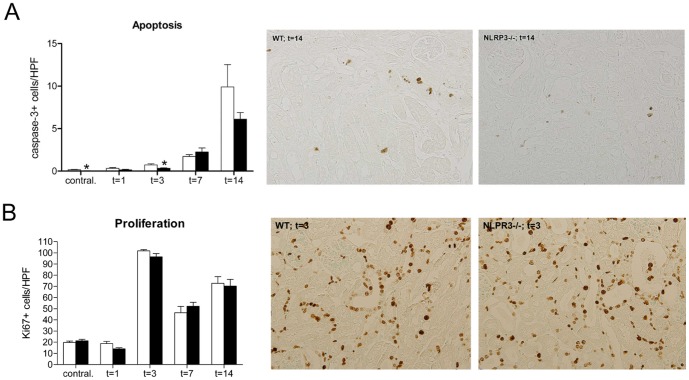
Nlrp3 deficiency mildly reduces number of apoptotic but not proliferating tubular epithelial cells following UUO. The number of (A) apoptotic (active caspase 3^+^) TECs was mild but significantly lower in contralateral and 3-day obstructed kidneys of Nlrp3−/− (black bars) mice compared to wild type mice (white bars), whereas at other time points no differences were observed. (B) The number of proliferating TECs was comparable between wild type and Nlrp3−/− mice at all time points. Data are mean±SEM of 7 mice per group. Contral.  =  contralateral kidneys of mice subjected to 14 days of UUO. *:p<0.05. The panels show representative microphotographs of renal tissue sections, magnification x200.

We additionally evaluated whether the observed differences in tubular injury and integrity between wild type and Nlrp3−/− mice could be ascribed to an effect of Nlrp3 deficiency on tubular apoptosis and proliferation. Despite increased tubular damage Nlrp3−/− mice had slightly less apoptotic TECs 3 days post-UUO (figure 5a), whereas at other time points no differences were observed. The number of proliferating TECs was comparable at all time points between wild type and Nlrp3−/− mice (figure 5b).

### Nlrp3 deficiency does not affect macrophage accumulation but impairs early renal IL1-β levels following UUO

**Figure 6:**
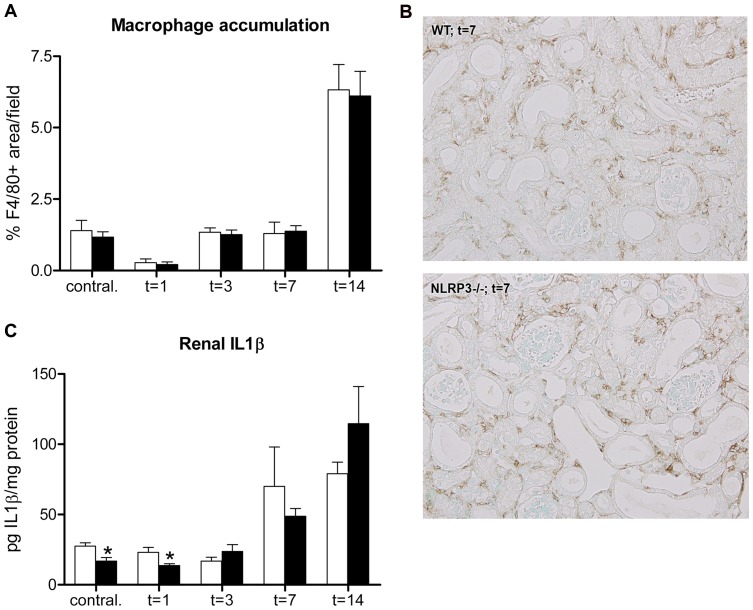
Nlrp3 deficiency reduces early renal IL1-β levels but does not affect macrophage accumulation following UUO. (A) Macrophages progressively accumulated in obstructed kidneys upon UUO and were comparable between wild type (white bars) and Nlrp3−/− (black bars) mice following UUO. (B) Representative microphotographs of macrophage accumulation in renal tissue sections of mice subjected to 7 days of UUO, magnification x200. (C) Nlrp3−/− mice displayed less renal IL1-β in their contralateral and obstructed kidneys after 1 day of UUO, whereas levels were comparable to wild type mice at later time points. Data are mean±SEM of 7 mice per group and indicated as pg IL1-β per mg protein. Contral.  =  contralateral kidneys of mice subjected to 14 days of UUO. *:p<0.05.

To determine whether Nlrp3 deficiency modulates tubulo-interstitial inflammation following UUO, macrophage accumulation and the levels of proinflammatory cytokines/chemokines IL1-β, KC, MCP-1 and TNF-α were analyzed in kidneys. Wild type mice displayed a progressive accumulation of macrophages in obstructed kidneys following UUO (figure 6A+B). Nlrp3 deficiency however did not influence macrophage accumulation. Renal IL1-β levels were significantly reduced in contralateral and obstructed kidneys (t = 1) of Nlrp3−/− mice compared to wild type mice, whereas levels at later time points did not differ (figure 6C). Nlrp3 deficiency did not influence renal concentrations of MCP-1, TNF-α or KC following UUO (figure S4).

### Nlrp3 deficiency does not affect renal fibrosis following UUO

**Figure 7:**
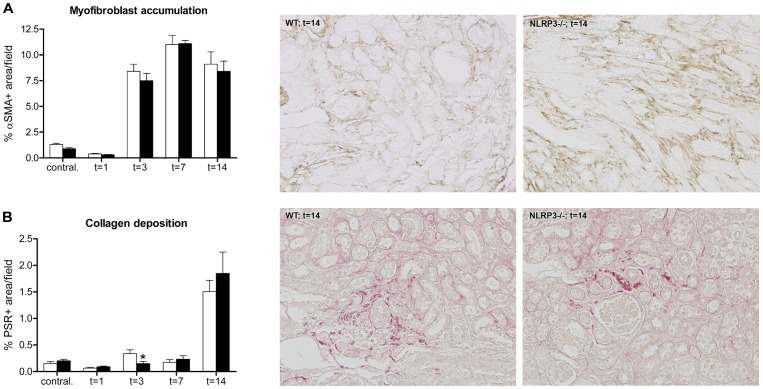
Nlrp3 deficiency does not affect myofibroblast accumulation and collagen deposition following UUO. Renal myofibroblast accumulation (A) and collagen deposition (B) in obstructed and contralateral kidneys of wild type (white bars) and Nlrp3−/− (black bars) mice following UUO. Nlrp3 deficiency did not affect the accumulation of myofibroblasts in kidneys following UUO (A). Except for a difference observed at 3 days post-UUO Nlrp3 deficiency did not influence renal collagen deposition following UUO (B). Data are mean±SEM of 7 mice per group. Contral.  =  contralateral kidneys of mice subjected to 14 days of UUO. *:p<0.05. The panels show representative microphotographs of renal tissue of mice subjected to 14 days of UUO, magnification x200.

Finally, we evaluated whether Nlrp3 deficiency affects renal fibrosis following UUO by analyzing the accumulation of myofibroblasts, total collagen deposition and renal concentrations of the profibrotic molecule Transforming Growth Factor-β (TGF-β). As expected, wild type mice displayed a progressive myofibroblast accumulation (figure 7A) and collagen deposition (figure 7B) in their obstructed kidneys following UUO. Nlrp3 deficiency did however not affect myofibroblast accumulation (figure 7A) or collagen deposition, except for a reduction observed at 3 days post-UUO (figure 7B). Moreover, Nlrp3 deficiency did not affect the renal concentrations of total TGF-β following UUO (figure S4).

## Discussion

The inflammasome component Nlrp3 is constitutively expressed in the kidney and clearly enhanced following acute and chronic kidney disease [9], [10], [11], [14](this study). Nlrp3 contributes to renal inflammation through its classical function of caspase-1-mediated cleavage and subsequent release of effector cytokines. Additionally, recent studies suggest non-canonical effects for Nlrp3 in the pathophysiology of acute and chronic kidney injury [15], [16]. Apparently, Nlrp3 seems to have effects on tissue homeostasis and inflammation that are beyond its classical role in cytokine maturation. Hence, in this study we investigated whether Nlrp3 exert additional functions in the kidney during the initial phase of progressive renal injury by subjecting wild type and Nlrp3−/− mice to unilateral ureter obstruction (UUO).

We observed that Nlrp3, but not ASC mRNA expression progressively increased following UUO, which is in agreement with previous results [10]. Our data suggest that in contrast to endothelial cells, TECs and myofibroblasts in particular are an important source of non-immune renal Nlrp3 and ASC expression. Interestingly, Nlrp3 attenuates renal damage following UUO, as reflected by significant enhanced levels of tubular injury in obstructed kidneys of Nlrp3−/− mice 1, 3 and 7 days post-UUO compared to wild type mice. In accordance, Nlrp3−/− mice displayed enhanced mRNA expression of the renal injury biomarker NGAL 1 day post-UUO [21], [22]. Moreover, tubular dilatation and brush border disappearance was apparent in Nlrp3−/− obstructed kidneys as reflected by an altered expression pattern of the proximal tubular brush border marker SGLT1 [23] **.** After 14 days no differences in renal injury could be found anymore as tubular injury was at maximum in both groups. In contrast to these results, we [9] and others [17] previously found that the Nlrp3 inflammasome enhanced acute kidney injury and dysfunction following ischemia reperfusion injury. These opposite effects of Nlrp3 deficiency in acute and progressive renal injury are also seen in mice deficient for TLR4 [6], [24]. Obviously, signaling via pattern recognition receptors can lead to a profoundly different outcome of local injury and inflammation during acute (ischemic) or chronic (UUO) renal injury, and implicates that targeting these receptors for the treatment of renal diseases requires careful consideration.

Additionally, a major characteristic observed in obstructed kidneys of Nlrp3−/− mice was prominent interstitial edema, possibly as a result of increased intratubular pressure-induced mechanical stretch and enhanced vascular and/or tubular permeability. Indeed, Nlrp3−/− mice demonstrated more tubular dilatation and a reduced mRNA expression of claudin-1, -2, -5 and VE-Cadherin 1 day post-UUO compared to wild type mice. Claudin-1 and -2 exhibit a specific expression pattern in the kidney and are thought to be essential in tight junction formation to establish close connections between epithelial cells, thereby maintaining cell polarity and tubular integrity [25], [26], [27]. On the contrary, Claudin-5 and VE-Cadherin are specifically expressed in endothelial cells to maintain vascular barrier functions and regulate endothelial permeability [28], [29]. Together, this suggests that Nlrp3 protects against progressive renal injury by directly or indirectly mediating expression of renal epithelial and endothelial intercellular junction molecules that leads to a better preservation of vascular barrier and epithelial integrity in response to mechanical stretch. In line, it has been shown that stretch adversely affects tight junction structures on epithelial cells, thereby modulating epithelial permeability [30], [31]. The relevance of the subtle differences in Claudin-1 and -2 expression observed 14 days post-UUO remain unclear and need to be investigated in more detail in the future.

To further characterize the non-canonical effect of Nlrp3 and to verify whether Nlrp3 affects vascular permeability we quantified interstitial Dextran accumulation early upon UUO. High molecular weight Dextran cannot be filtered by intact glomeruli, consequently retains in the vasculature and is therefore an excellent marker for blood vessel wall integrity [32], [33], [34]. Interestingly, vascular leakage was significantly elevated in Nlrp3−/− obstructed kidneys 1 day post-UUO. In line with reduced mRNA expression of renal Claudin-5 and VE-Cadherin, vascular leakage was also elevated in the contralateral kidneys of Nlrp3−/− mice. Together, this indicates that Nlrp3 affects vascular barrier integrity in a model of UUO, thereby preventing the development of renal interstitial edema. The limited contribution of endothelial cells to renal Nlrp3 expression (own data and [15], [35], [36]) might point towards an indirect effect of Nlrp3 on vascular integrity and permeability through modulation of the local chemical and mechanical microenvironment of endothelial cells. An alternative explanation might be that endothelial cells are activated through the uptake of monocytic cell-derived microparticles containing inflammasome components [37]. Whether this occurs in renal vasculature and its consequences on permeability upon injury remains unclear and should be investigated in future. The decreased epithelial barrier function in Nlrp3−/− mice was not associated with increased apoptosis of renal epithelial cells and/or decreased cell proliferation in our study.

The major canonical effect of Nlrp3 is cytokine maturation [38]. Although Nlrp3 deficiency mildly reduced the amount of early renal IL1-β protein, these levels do not rise above the contralateral kidney. This difference moreover disappeared while injury progresses, proposing redundancy through other members of the Nlrp protein family that may form additional IL1-β activating multi-protein complexes. The concentrations of renal KC, MCP-1 and TNF-α were not affected by absence of Nlrp3. In contrast to the pivotal role we demonstrated for Nlrp3 in renal inflammation following acute ischemic injury [9], macrophage accumulation was not altered by Nlrp3 deficiency in the present study. In agreement, deficiency of either IL1-β or IL1-R1, both functioning downstream of inflammasome activation, does not affect macrophage accumulation following UUO [39].

We further investigated whether Nlrp3 influenced the progression of renal fibrosis. However, no differences were observed in TGF-β levels, accumulation of myofibroblasts or collagen deposition in obstructed kidneys of Nlrp3−/− mice following UUO, except a slight but significant decrease 3 days post-UUO. In line, IL1-β deficiency did not affect accumulation of myofibroblasts or collagens in kidneys following obstruction-induced renal fibrosis [39], whereas transgenic mice with neutralized IL-18 by IL-18 binding protein exhibited reduced collagen deposition and myofibroblast accumulation during UUO, without altering the concentrations of TGFβ [40]. Together, it can thus be proposed that development of progressive renal fibrosis is primarily mediated via IL-18, while the role of upstream Nlrp3 is only minor. Although, neutralizing IL-18 activity via IL-18BP may have pleiotropic effects that could interfere with disease outcome.

In accordance to our data, Vilaysane *et al.* showed that Nlrp3 deficiency reduced numbers of apoptotic cells and renal IL1-β levels following UUO [10]. In contrast, they observed less renal injury in Nlrp3−/− kidneys 2 weeks post-UUO as determined by scoring the percentage of cortical tubular necrosis as injury parameter. The reason that we could not confirm these effects for Nlrp3 on fibrosis, inflammation and injury at day 14 is unclear but could merely reflect differences in the model used. While we [6], [8] and others [3], [41], [42], [43], [44], [45], [46] observed that the major pathological change associated with urinary tract obstruction is the loss of renal tubular cells via apoptosis, Vilaysane *et al.* found that renal injury is particularly reflected by high levels of cortical tubular necrosis (up to 80%) [10].

In conclusion, our results reveal a novel non-canonical effect of Nlrp3 in the maintenance of renal integrity and protection against the early effects of UUO-induced progressive renal injury. On the contrary, absence of Nlrp3 only slightly affects renal inflammatory processes following UUO-induced renal injury.

## Supporting Information

Figure S1 **Renal cell-specific expression of inflammasome components.** Expression of Nlrp3 (A), ASC (B) and caspase-1 (C) mRNA in wild type primary tubular epithelial cells (TECs), myofibroblasts or endothelial cells stimulated with (+) or without (−) 10 ng/ml TGFβ for 24 hours. In addition, bone-marrow-derived macrophages (BMDM; LPS+IFNγ stimulated) were included as a positive control. Nlrp3, ASC and caspase-1 mRNA expression in non-immune renal cells was primarily observed in TECs and myofibroblasts. Data are mean±SEM of 2−4 samples per group.
(TIF)

Figure S2 **Nlrp3 deficiency results in tubular dilatation and diffuses proximal tubular brush borders following UUO.** Representative microphotographs of renal SGLT1 expression following UUO, specifically expressed at the brush border of proximal tubular compartment. Renal SGLT1 expression pattern is markedly altered in Nlrp3−/− mice compared to wild type kidneys after 1 day of UUO. No differences were observed between contralateral kidneys of wild type and Nlrp3−/− mice 1 day after UUO. Representative photographs from n = 6 mice/group (magnification x200).
(TIF)

Figure S3 **Nlrp3 deficiency does not affect basal renal expression of tubular or vascular adhesion components.** Expression of Claudin-1 (A), Claudin-2 (B), Claudin-5 (C) and VE-Cadherin (D) mRNA was comparable between kidney tissue of naïve wild type (white bars) and Nlrp3−/− (black bars) mice. Data are mean±SEM of 6 mice per group.
(TIF)

Figure S4 **Nlrp3 deficiency does not affect proinflammatory or profibrotic cytokine and chemokine levels following UUO.** Renal levels of proinflammatory cytokines and chemokines KC (A), MCP1 (B) and TNFα (C) were comparable in homogenates of wild type (white bars) and Nlrp3−/− (black bars) kidneys. Total renal levels of the profibrotic molecule TGFβ (D) were similar in obstructed and contralateral kidneys of wild type and Nlrp3−/− mice. Data are mean±SEM of 7 mice per group and indicated as pg per mg protein. Contral.  =  contralateral kidneys of mice subjected to 14 days of UUO. *:p<0.05.
(TIF)
