# Supplementary figures and images for: Nlrp3 Prevents Early Renal Interstitial Edema and Vascular Permeability in Unilateral Ureteral Obstruction
Source: PLoS One. 2014 Jan 15;9(1):e85775. doi: 10.1371/journal.pone.0085775 (PMC3893260; doi:10.1371/journal.pone.0085775)

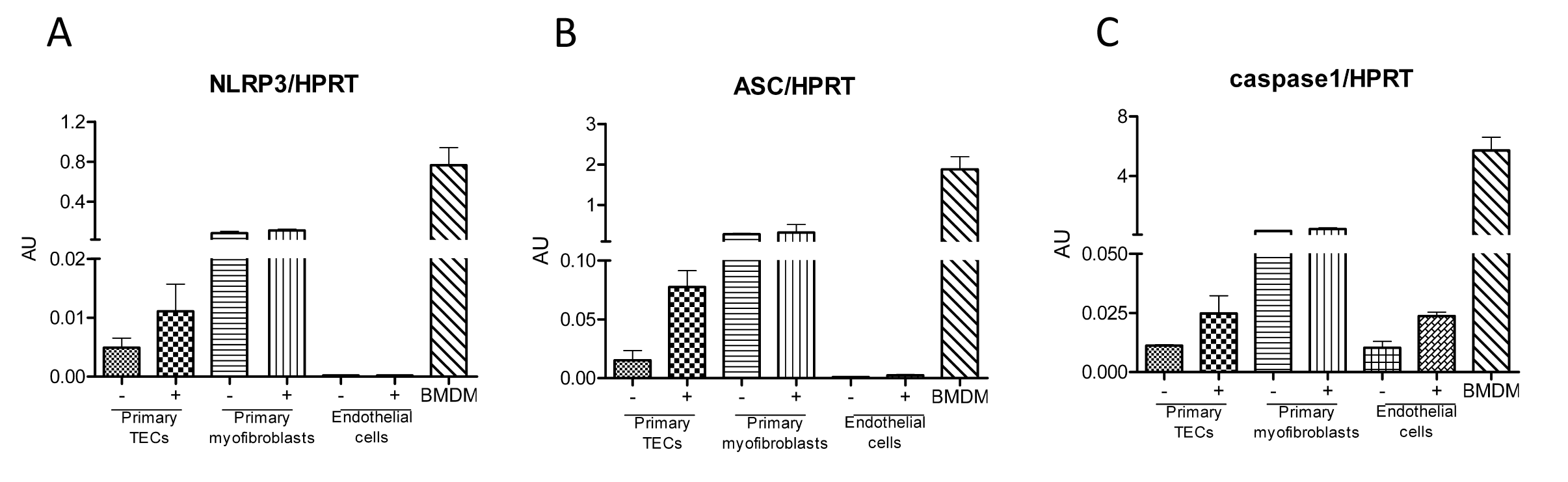

Supplement: Figure S1 — Renal cell-specific expression of inflammasome components. Expression of Nlrp3 (A), ASC (B) and caspase-1 (C) mRNA in wild type primary tubular epithelial cells (TECs), myofibroblasts or endothelial cells stimulated with (+) or without (−) 10 ng/ml TGFβ for 24 hours. In addition, bone-marrow-derived macrophages (BMDM; LPS+IFNγ stimulated) were included as a positive control. Nlrp3, ASC and caspase-1 mRNA expression in non-immune renal cells was primarily observed in TECs and myofibroblasts. Data are mean±SEM of 2−4 samples per group. (TIF) [file pone.0085775.s001.tif]

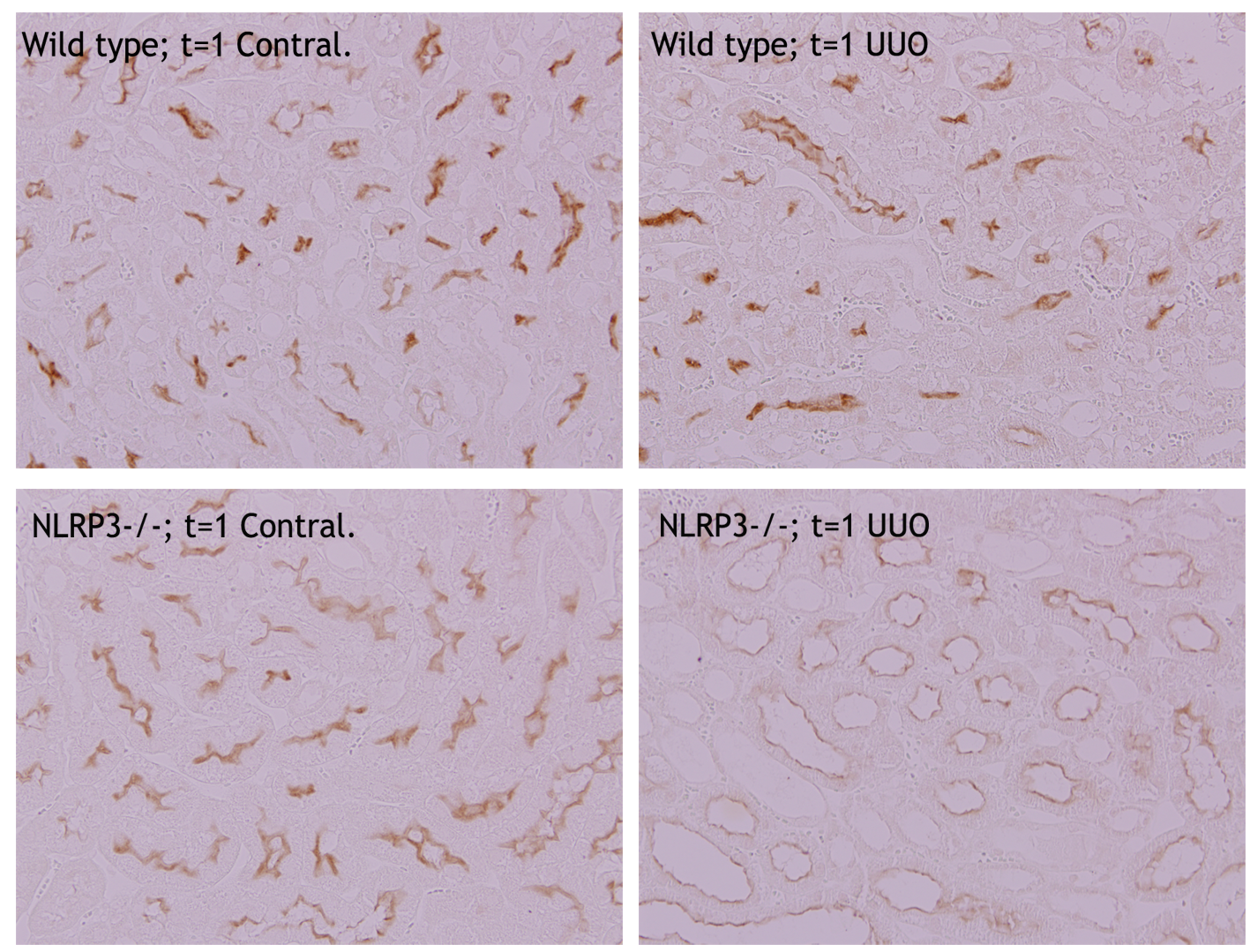

Supplement: Figure S2 — Nlrp3 deficiency results in tubular dilatation and diffuses proximal tubular brush borders following UUO. Representative microphotographs of renal SGLT1 expression following UUO, specifically expressed at the brush border of proximal tubular compartment. Renal SGLT1 expression pattern is markedly altered in Nlrp3−/− mice compared to wild type kidneys after 1 day of UUO. No differences were observed between contralateral kidneys of wild type and Nlrp3−/− mice 1 day after UUO. Representative photographs from n = 6 mice/group (magnification x200). (TIF) [file pone.0085775.s002.tif]

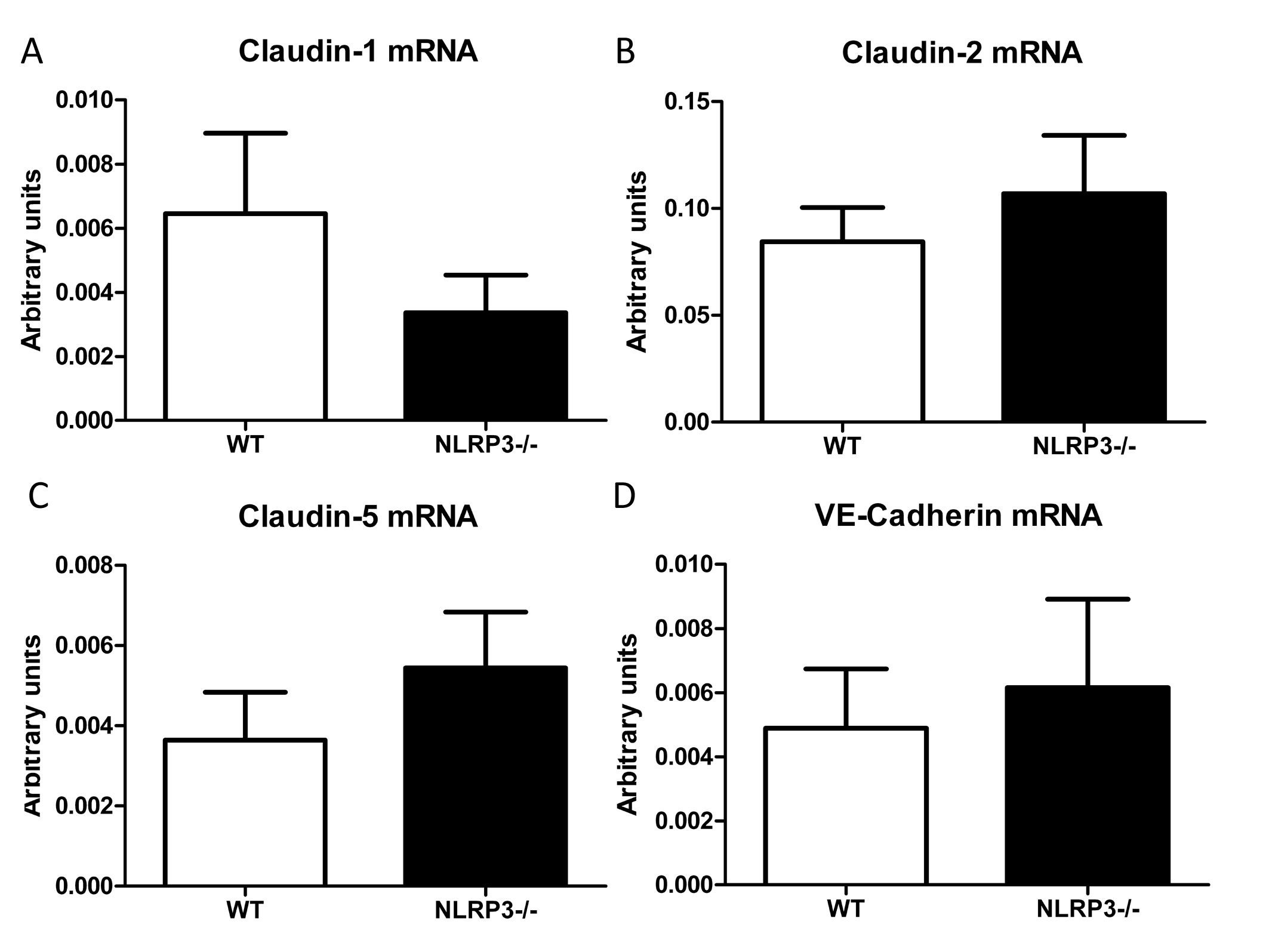

Supplement: Figure S3 — Nlrp3 deficiency does not affect basal renal expression of tubular or vascular adhesion components. Expression of Claudin-1 (A), Claudin-2 (B), Claudin-5 (C) and VE-Cadherin (D) mRNA was comparable between kidney tissue of naïve wild type (white bars) and Nlrp3−/− (black bars) mice. Data are mean±SEM of 6 mice per group. (TIF) [file pone.0085775.s003.tif]

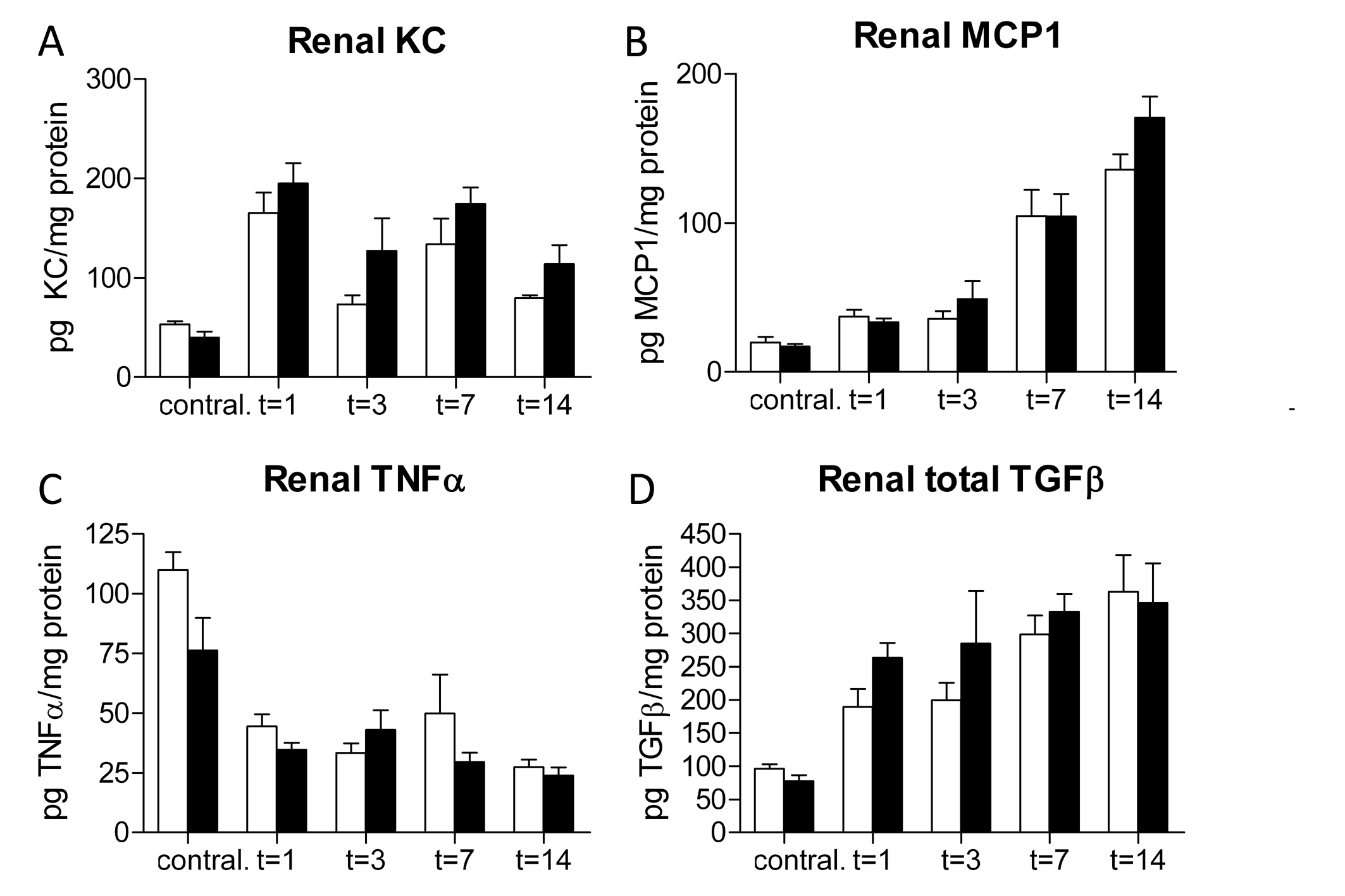

Supplement: Figure S4 — Nlrp3 deficiency does not affect proinflammatory or profibrotic cytokine and chemokine levels following UUO. Renal levels of proinflammatory cytokines and chemokines KC (A), MCP1 (B) and TNFα (C) were comparable in homogenates of wild type (white bars) and Nlrp3−/− (black bars) kidneys. Total renal levels of the profibrotic molecule TGFβ (D) were similar in obstructed and contralateral kidneys of wild type and Nlrp3−/− mice. Data are mean±SEM of 7 mice per group and indicated as pg per mg protein. Contral. = contralateral kidneys of mice subjected to 14 days of UUO. *:p<0.05. (TIF) [file pone.0085775.s004.tif]
